# Vitamin D and Beta-Glucans Synergically Stimulate Human Macrophage Activity

**DOI:** 10.3390/ijms22094869

**Published:** 2021-05-04

**Authors:** Loredana Bergandi, Giulia Apprato, Francesca Silvagno

**Affiliations:** Department of Oncology, University of Torino, Via Santena 5 bis, 10126 Torino, Italy; loredana.bergandi@unito.it (L.B.); giulia.apprato@edu.unito.it (G.A.)

**Keywords:** vitamin D, beta-glucans, human macrophage, immunostimulation, interleukin-8, vesicle acidification, vacuolar ATPase, mitochondrial metabolism

## Abstract

Vitamin D and beta-glucans are both immunostimulants. Vitamin D exerts its beneficial effects on many components of the immune system. In macrophages, the hormone modulates both phagocytic activity and cytokine production; therefore, it plays an important role in mediating the innate immune response to infection. The immunomodulatory properties of beta-glucans are attributed to the ability of these fungal cell wall polysaccharides to bind to different receptors expressed on the cell surface of phagocytic and cytotoxic innate immune cells, including monocytes and macrophages. The intracellular signaling pathways activated by beta-glucans lead to enhanced phagocytosis and cytokine response. In this study we investigated the possible potentiation of immunomodulatory properties of the combined treatment with vitamin D and beta-glucans. The effects of 100 nM 1,25-dihydroxyvitamin D3 or 100 µg/mL beta-glucans were evaluated in human macrophages in terms of cytokine production, intracellular vesicle acidification and changes in energy metabolism, three hallmarks of macrophage antimicrobial activation. We found that all the analyzed parameters were enhanced by the co-treatment compared to the response to single molecules. The results of this study support the validity of a novel therapeutic approach that could boost the immune response, taking advantage of the synergy between two natural compounds.

## 1. Introduction

Macrophages are immune cells specialized in the phagocytosis and further digestion of pathogens, cellular debris, senescent and cancer cells as well as other foreign substances. Given their innate immunity, they are involved in the immediate response but also in the initiation of the induced response, recruiting lymphocytes through the release of a specific set of chemokines and cytokines [1]. These two classes of molecules act as chemoattractants and therefore are produced and secreted to recruit other cells of the innate and adaptive immune system. Among the several chemokines produced, IL-8 plays a fundamental role. It recruits T-cells, neutrophils and basophils, which through chemotaxis arrive to the site of infection where they explicate their function [2].

Macrophages bind several microbial molecules, known as pathogen-associated molecular patterns (PAMPs), such as lipopolysaccharide (LPS) and the polysaccharides beta-glucans. PAMPs are recognized by pattern recognition receptors (PRRs), receptors expressed on the surface of macrophages [1]. PRRs are evolutionarily conserved germline-encoded receptors and are divided into different families. Toll-like receptors (TLRs) are PRRs involved in the binding of microbial moieties and in triggering the further signaling through several intracellular pathways.

Upon binding, the pathogen is trapped into the phagosome, a vesicle created by the macrophage in order to phagocyte the invader. The strongly acidic pH and the enzymes present in the phagolysosome mediate the pathogen digestion. The phagosomal maturation process is a key step for a functional macrophage and is enabled by the recruitment of vacuolar-type ATPase (V-ATPase) [3]. This enzyme pumps protons through ATP-dependent counter-gradient activity to acidify the vesicles. Acidification is central for macrophage activity, and defects in this mechanism lead not only to macrophage malfunction but also to a scarce activation of intracellular pathways driving the production of immunomodulatory molecules. Among the several molecules known to induce the V-ATPase expression, vitamin D plays an important role and has been investigated mainly in osteoclasts, tissue specific macrophage-like cells [3].

Calcitriol or 1,25-dihydroxycholecalciferol is the active form of vitamin D and is well known for its role in mineral and skeletal homeostasis [4]. However, a plethora of studies have demonstrated that vitamin D plays a variety of different roles, including the modulation of immune responses [5]. The expression of vitamin D receptor (VDR) and the activity of 25-hydroxyvitamin D-1 hydroxylase (CYP27B1), which is the enzyme that catalyzes the synthesis of the active form of vitamin D [6,7], have been described in inflammatory cells. Namely, both VDR, which mediates vitamin D effects, and CYP27B1 are expressed in macrophages, monocytes and dendritic cells [6,8]. In particular, CYP27B1 activity in macrophages seems to be highly responsive to stimulation with immunoactivators such as interferon-γ (IFN-γ) and LPS [6].

The immunomodulatory properties of vitamin D have been assayed in different immune cell types. Carlberg and colleagues have performed a genome-wide analysis of VDR binding sites in THP-1, a human monocyte cell line, demonstrating that active vitamin D administration causes the upregulation of IL-8 gene and other chemokines too [9]. Other studies confirmed that vitamin D in the presence of LPS facilitates IL-8 production in neutrophils boosting the immune response [5]. Although its immunomodulatory activity appears evident, the details of vitamin D signaling are still not clear. Interestingly, a recent pathway enrichment analysis carried out in primary cultures of dendritic cells, monocytes and THP-1 cells has concluded that vitamin D seems to be a positive regulator of mitochondrial metabolism in immune cells, by inducing a significant upregulation in genes related to the electron transport chain, the oxidative phosphorylation and the tricarboxylic acid (TCA) cycle [7]. Despite these new insights, the overall mechanism of immunomodulation triggered by vitamin D has not been completely elucidated.

β-glucans are a class of PAMPs exerting an immunomodulatory activity similar to the one exerted by vitamin D. They are one of the major constituents of some fungi, bacteria, yeast and plant cell walls. They are a somewhat heterogenous family of polysaccharides characterized by a backbone of β-(1-3)-linked D-glucose units with β-(1-6)-linked branches of various length [10]. These polysaccharides are biological response modifiers, and thus they are employed as anti-tumor and anti-infective drugs. They have been demonstrated to be able to protect against bacterial and viral infections and also to prevent cancer progression if administered in combination with monoclonal antibodies or chemotherapy [10,11,12]. 

The immunomodulatory activity of β-glucans consists of the enhancement of macrophages, granulocytes, monocytes and dendritic cells’ cytotoxic activity and inflammatory cytokine production [10,13]. Notwithstanding the interest in these molecules, β-glucans’ mechanism of action remains to be elucidated. However, it has been suggested that they exert their activity through the binding of detectin-1 and TLRs, triggering macrophage activation. TLR binding is known to induce several signaling pathways resulting in the activation of myeloid differentiation factor 88 (MyD88), nuclear factor-κB (NF-κB) and mitogen-activated protein kinase (MAPK) pathways, culminating in the production of pro-inflammatory cytokines [10,14,15]. In addition, TLR signaling seems to require the acidification of the vesicles containing the pathogen [14]. 

In this study we investigated the synergistic effects of the active form of vitamin D (hereinafter referred to as vitamin D) and β-glucans as immunomodulatory molecules in two human macrophage cell lines, THP-1 and U-937. Macrophage activity was evaluated in terms of (i) cytokine production; in particular, we measured IL-8, a well-established marker of macrophage activity; (ii) intracellular vesicle acidification and (iii) changes in the energy metabolism. The molecular mechanisms underlying the synergy were characterized. 

## 2. Results

### 2.1. Vitamin D and Beta-Glucans Stimulate the Synthesis and Secretion of IL-8 in Human Macrophages

**Figure 1:**
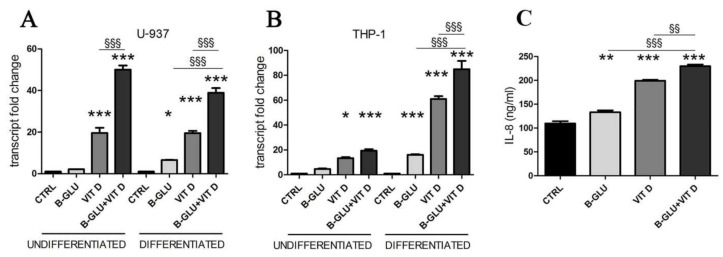
Vitamin D and beta-glucans stimulate IL-8 expression and secretion. Two cell lines were differentiated to macrophages by TPA treatment or used as undifferentiated monocytes. After 24 h of treatment with 100 µg/mL beta-glucans (B-GLU) and 100 nM vitamin D (VIT D) the transcript levels of IL-8 were measured by real-time PCR. (**A**) U-937 cells; (**B**) THP-1 cells. (**C**) The levels of IL-8 were measured in the culture medium of differentiated U-937 cells. Results are means ± SEM. * *P* < 0.05, ** *P* < 0.01 and *** *P* < 0.001 vs. ctrl; ^§*§*^ *P* < 0.01 and ^§*§§*^ *P* < 0.001.

Given the immunostimulating potential of the two molecules, we investigated their effects on human macrophages, as single treatment or in combination. We used two models of human monocytes easily differentiated into macrophages, the U-937 and THP-1 cell lines. Differentiation was carried out by a 24-h treatment with 12-O-tetradecanoylphorbol-13-acetate (TPA), and the effect of beta-glucan and vitamin D was tested after 24 h of treatment of both undifferentiated and differentiated cells. As a read-out of immunostimulation, we assayed the transcription of IL-8, a cytokine typically produced by activated macrophages. The real-time PCR analysis revealed the increased expression of the cytokine upon single treatments, as shown in Figure 1. Vitamin D was effective in all cells, whereas beta-glucans induced the transcription only in differentiated cells. Interestingly, the combination further enhanced the transcription of IL-8 in most cells (Figure 1); the effect was evident in the differentiated phenotype of both cell lines, whereas in undifferentiated cells the result of co-stimulation was discrepant. The secretion of IL-8 detected in culture medium of differentiated cells confirmed the increased expression of the cytokine (Figure 1C). Therefore, further analysis was carried out on differentiated macrophages, which in any case are the players of the innate immune response in tissues. In this first set of experiments we found the evidence of an amplified immunomodulatory activity of beta-glucans and vitamin D when co-administered to macrophages, and we decided to investigate the molecular mechanisms underlying the synergy.

### 2.2. Vitamin D and Beta-Glucans Stimulate the Intracellular Vesicle Acidification of Human Macrophages

**Figure 2:**
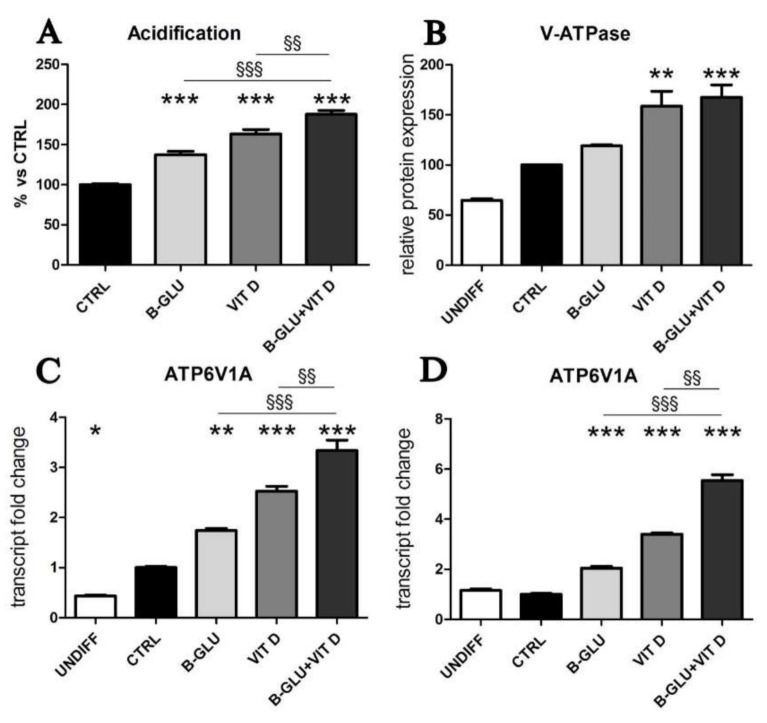
Vitamin D and beta-glucans stimulate vesicular acidification. The two cell lines were differentiated to macrophages by TPA treatment or used as undifferentiated monocytes and were analyzed after 24 h of treatment with 100 µg/mL beta-glucans (B-GLU) and 100 nM vitamin D (VIT D). (**A**) In differentiated U-937 cells, acidification was measured as neutral red accumulation; (**B**) in undifferentiated (undiff) and differentiated and treated U-937 cells the levels of V-ATPase protein expression were evaluated by Western blotting; the transcription of subunit A of the V_1_ domain of V-ATPase (ATP6V1A) was quantified by real-time PCR in undifferentiated (undiff) and differentiated and treated cells: (**C**) U-937 cells and (**D**) THP-1 cells. Results are expressed relative to control and as the means ± SEM. * *P* < 0.05, ** *P* < 0.01 and *** *P* < 0.001 vs. ctrl; ^§§^ *P* < 0.01 and ^§*§§*^ *P* < 0.001.

**Figure 3:**
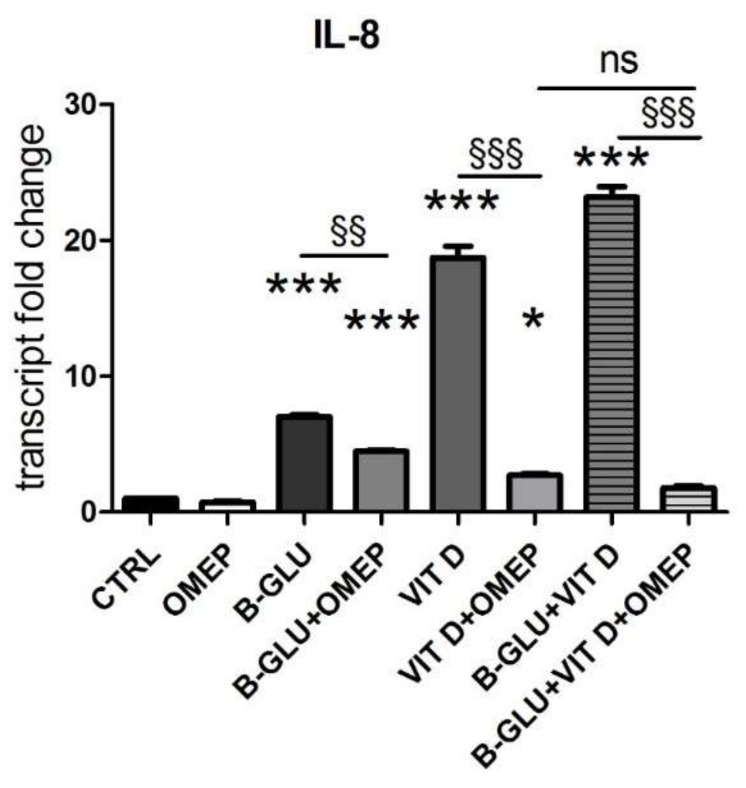
The immunomodulatory activity of beta-glucans and vitamin D is blocked by the inhibition of vacuolar acidification. U-937 cells were differentiated by TPA and were analyzed after 24 h of treatment with 100 µg/mL beta-glucans (B-GLU) and 100 nM vitamin D (VIT D) in the presence or absence of 200 µM omeprazole (OMEP). The transcript levels of IL-8 were measured by real-time PCR. Results are expressed relative to control and as the means ± SEM. * *P* < 0.05 and *** *P* < 0.001 vs. ctrl; ^§§^ *P* < 0.01 and ^§*§§*^ *P* < 0.001. ns: not significant.

Another marker of macrophage activation is represented by the acidification of phagosomal/lysosomal compartment. Both the killing of phagocyted microorganisms and the stimulation to produce chemoattractants recruiting the whole immune system rely on proper acidification of vesicles. Given its central role, organelle acidification is induced during differentiation along the monocyte/macrophage lineage [16]. With the aim of testing the effect of vitamin D and beta-glucans on the activation of macrophages, we measured acidification of differentiated cells by neutral-red accumulation assay (Figure 2A), and we analyzed the levels of vacuolar ATPase, the proton pump that lowers pH in intracellular compartments (Figure 2B–D). Acidification was found negligible in undifferentiated cells and was therefore quantified only in TPA-differentiated macrophages. V-ATPase expression was evaluated as protein levels by Western blotting, whereas transcription of the subunit A of the V_1_ domain of V-ATPase was evaluated by PCR analysis. In agreement with the scarce acidification, the undifferentiated cells showed low levels of V-ATPase expression. Data displayed in Figure 2 demonstrate that all the analyzed parameters were increased by single treatments and further enhanced by the cotreatment with the two molecules. The similar boosted response observed in the induction of both IL-8 expression and acidification upon combined treatment put forward the possibility of a central role for acidification in the synergic immunomodulatory activity of beta-glucans and vitamin D. In order to verify this causal link, we carried out a set of experiments in which the vesicular acidification was blocked by the proton pump inhibitor omeprazole. In this experimental setting, we found that both beta-glucans and vitamin D were not able to fully induce IL-8 transcription, as shown in Figure 3. The abatement of induction was particularly noticeable when omeprazole was used together with the co-treatment. Despite the notable induction triggered by beta-glucans and vitamin D together, omeprazole reduced IL-8 levels to values similar to the effect of omeprazole and single vitamin D treatment. These data demonstrated that acidification was required to trigger the signaling pathway leading to cytokine production and that also the synergy between beta-glucans and vitamin D activity was strongly influenced by vesicle acidification.

### 2.3. Vitamin D and Beta-Glucans Stimulate the Mitochondrial Metabolism of Human Macrophages

**Figure 4:**
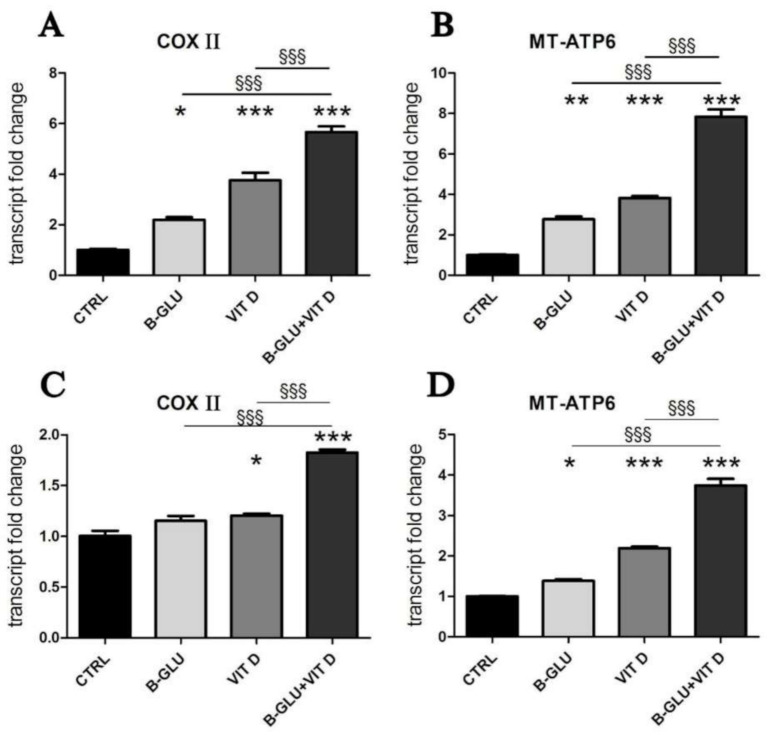
Vitamin D and beta-glucans stimulate the transcription of mitochondrial OXPHOS components. The two cell lines were differentiated to macrophages by TPA treatment. After 24 h of treatment with 100 µg/mL beta-glucans (B-GLU) and 100 nM vitamin D (VIT D), the transcript levels of cytochrome C oxidase subunit 2 (COX II) and mitochondrial ATP synthase F0 subunit 6 (MT-ATP6) were measured by real-time PCR. (**A,B**) Differentiated U-937 cells; (**C,D**) differentiated THP-1 cells. Results are means ± SEM. * *P* < 0.05 and *** *P* < 0.001 vs. ctrl; ^§*§§*^ *P* < 0.001.

In our search of molecular mechanisms responsible for the synergic immunomodulation exerted by beta-glucans and vitamin D, we wondered which metabolic conditions would support the optimal acidification and the consequent signaling. V-ATPase couples the energy of ATP hydrolysis to proton transport across intracellular membranes. Intracellular ATP is mainly produced by the mitochondrial respiratory activity; therefore, we decided to investigate the activation of the mitochondrial respiratory chain and oxidative phosphorylation (OXPHOS). As shown in Figure 4, in both cell lines we found that the transcription of the respiratory chain and ATP synthase subunit was induced by single treatments, and again the increase was enhanced by the combination of beta-glucans and vitamin D, in agreement with the observed effects on IL-8 transcription and acidification. By contrast, the stimulation of the respiratory chain did not alter the basal levels of ROS (reactive oxygen species), a common byproduct of respiratory activity (Figure S1). We concluded that the intracellular detoxification systems probably neutralized any radical species produced.

Altogether, the results of the study pointed toward a potentiation of the energy production necessary to sustain the ATP-dependent acidification process and translate the convergent signaling of beta-glucans and vitamin D. 

## 3. Discussion

Both vitamin D and beta-glucans are known immunomodulators. Their mechanisms of action and signaling pathways are apparently different, as reported so far by many studies; however, their combination could further activate the immune response, and this possible beneficial effect was never investigated before.

In this study we worked on two lines of monocyte/macrophage cells. We chose this model due to the importance of macrophages as a first line of defense against microbial invasion and due to the central role of the innate immune system in recruiting several adaptive immunity players. Some details of the effects exerted both by vitamin D and beta-glucans on monocyte/macrophage lineage were reported by previous studies and were mainly focused on signaling culminating in transcriptional modulation. Beta-glucans activate macrophages by an intracellular signaling starting from surface receptors such as TLRs and mediated by MAPK and NF-kB nuclear signaling, which leads to the production of several cytokines [13]. On the other hand, vitamin D is a well-known immunomodulator of macrophage activity, since it exerts a transcriptional control over several proteins critical for macrophage bactericidal function, upregulating for example the expression of cathelicidin and β-defensin 2 in phagocytes [17,18] and modulating cytokine production [19]. In this study, we were interested in verifying the possible reciprocal potentiation of their effects, finding the processes in which the two signaling pathways could converge.

First, we tested the transcription and secretion of IL-8, a chemotactic factor that attracts and activates neutrophils, basophils and T-cells, and we considered IL-8 modulation as a read-out of immunostimulation in our experimental model. We found that mainly the differentiated macrophages responded to single stimulations, and even more to the combination of vitamin D and beta-glucans. Whereas the induction caused by single stimulation was expected, since IL-8 was reported among the downstream targets of both vitamin D [9] and beta-glucan activity [20,21], the enhancement of the effect exerted by the two molecules together was a novel observation, which prompted further investigation. 

Seeking a convergence of the two signaling pathways, we focused on vesicular acidification. Due to an induced transcription of V-ATPase [22], acidification is increased during macrophage differentiation, and it is fundamental in many specialized functions of macrophages, such as proper phagocytic and killing activity and intracellular movements of vesicles [23]. We tested the modulation of the acidification and its causal mechanisms in differentiated human macrophages treated with vitamin D and beta-glucans. Indeed, vesicle acidification seemed a common and central step for both stimuli; both acidification and the expression of the transporter mainly involved in the maintenance of vacuolar acid pH were not only induced by single treatments, a novel observation in itself, but also most interestingly were greatly enhanced by the co-treatment. Vitamin D is a known inducer of V-ATPase at the transcriptional level, since its promoter contains potential vitamin D responsive elements [3]. The induction of V-ATPase expression and activity has been reported by studies investigating the transcriptional activity of vitamin D in osteoclasts [24,25], in agreement with our findings; our study adds novel observations in human macrophages. On the other hand, nothing was known about a similar control exerted by beta-glucans; therefore, these novel interesting data deserve further future investigation. 

Acidification is involved not only in bactericidal function of phagocytes, but also it is important in generating the intracellular signaling that leads to cytokine production. TLRs are present both in plasmamembrane and in the endosomal acid compartment; from the latter, upon activation, they communicate the microbial invasion to the nuclear compartment, eliciting the immune response [14]. The impact of acidification on cytokine production, modulated by the double stimulation, was verified when we discovered that inhibiting V-ATPase and consequently blocking acidification abated the immunostimulatory activity of vitamin D and beta-glucans together. The inhibitor of V-ATPase omeprazole affected only partially the expression of IL-8 induced by beta-glucans; therefore, it is reasonable to assume that the signaling triggered by beta-glucans was mediated both by vesicle and plasmamembrane receptors. Similarly, the inhibition of acidification did not block completely the effect of vitamin D on IL-8 expression, suggesting that in these cells the hormone was modulating IL-8 through a vesicle-mediated mechanism of action and in addition it was exerting a direct transcriptional control, as previously reported [9]. The results of this study suggest that the acidification of vesicles is essential for beta-glucan signaling pathways, and it is favored by the increase of ATPase induced by vitamin D. 

Furthermore, another interesting consideration arises from this study. In the presence of omeprazole, the residual vesicle-independent modulation of IL-8 must be exerted directly on transcription, by VDR and by NF-kB-dependent beta-glucan signaling. The data presented in this study suggest that in co-treatment with omeprazole and beta-glucans, not only does vitamin D not potentiate beta-glucan activity because the acidification is blocked, but rather VDR could inhibit NF-kB nuclear activity. Indeed, several studies have demonstrated the interplay between the two transcription factors [26,27,28]. In accordance with this hypothesis, we found that the effect of omeprazole on co-stimulation with beta-glucans and vitamin D was similar to the effect of omeprazole on single treatment with vitamin D because the nuclear signaling of beta-glucans through NF-kB could be impaired. The possibility that vitamin D could either enhance the inflammatory antimicrobial activity or moderate the nuclear pathways in different cellular settings opens interesting perspectives and warrants further studies on the multiple effects exerted by vitamin D depending on macrophage acidification and activation. The acidification of intracellular organelles occurs through an active, energy consuming, counter-gradient proton transport. The vacuolar ATPase is a proton pump that couples ATP hydrolysis to proton transport in the intracellular lysosomal and phagolysosomal compartments; the ATP required for its activity is mainly produced by mitochondrial oxidative phosphorylation (OXPHOS). ATP shortage resulting from mitochondrial dysfunction has been proposed as the cause of impaired lysosomal acidification [29]. Moreover, abundant mitochondrial activity is required in cells particularly active in acidification processes. For example, osteoclasts and epididymal clear cells are members of the family of “mitochondria-rich” cells, which have common characteristics including abundant mitochondria, high endocytic activity and abundant V-ATPase in intracellular vesicles and plasma membrane [30]. Based on these observations, we hypothesized that the modulation of mitochondrial energy production could be involved in the mechanisms underlying the synergic immunomodulatory activity of beta-glucans and vitamin D. Indeed, the transcription of key subunits of the respiratory chain and OXPHOS was upregulated by the two molecules in both differentiated cell lines, suggesting a role for mitochondrial ATP production in acidification and immunomodulation of macrophages. Our experimental evidence supports the increased mitochondrial metabolism consequent to vitamin D treatment, which was described in a recent bioinformatic pathway analysis also carried out on THP-1 cells. This study found that vitamin D increased the expression of genes associated with electron transport chain complexes and oxidative phosphorylation pathway [7]. The analysis performed in the present study confirms the fundamental role for active vitamin D as a pivotal regulator of immunometabolism.

**Figure 5:**
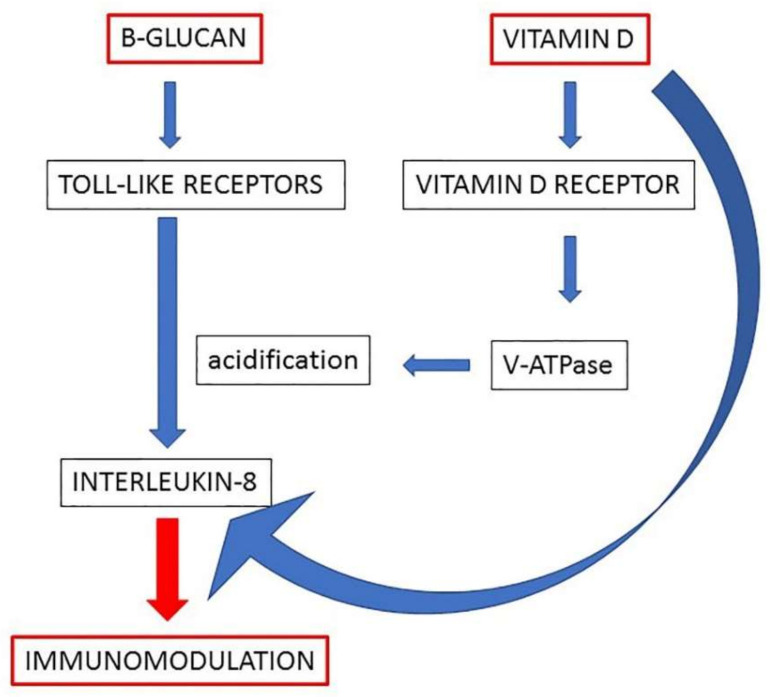
A working model of the molecular mechanisms underlying the effects of beta-glucans and vitamin D on immunomodulation of macrophages. The enhanced acidification represents the central step by which the two molecules can potentiate the production of IL-8 in macrophages.

Altogether, the results of this study brought forward the evidence of a common signaling pathway, which could explain the synergic immunomodulatory activity of beta-glucans and vitamin D. The proposed model, depicted in Figure 5, predicts that both molecules increase vesicular acidification by increasing V-ATPase expression and energy at its disposal and enhance the signaling that leads to the production of immunomodulatory cytokines. The encouraging results of this in vitro study warrant further investigation by clinical trials in order to confirm the efficacy of the co-treatment with beta-glucans and vitamin D when the immune system needs to be potentiated.

## 4. Materials and Methods 

### 4.1. Cell Culture and Treatments

The pro-monocytic human myeloid leukemia cell line U-937 and the human monocytic cell line THP-1 were purchased from American Type Culture Collection (ATCC, Manassas, VA 20110, USA) and were cultured in RPMI medium supplemented with 10% fetal bovine serum and 1% antibiotics (penicillin-streptomycin) at 37 °C in humidified 5% CO_2_ atmosphere. Differentiation of both U-937 and THP-1 cells was induced using 15 ng/mL 12-O-tetradecanoylphorbol-13-acetate (TPA) for 24 h [31,32,33,34]. The two cell lines are the most commonly used models of human monocytes, easily differentiated in macrophages by TPA. U-937 and THP-1 cells show a large, round single-cell morphology; nearly all cells start to adhere to culture plates and differentiate into macrophages after exposure to TPA [35,36]. Undifferentiated and differentiated U-937 and THP-1 cells were treated with 100 nM 1,25(OH)_2_D_3_ or 100 µg/mL beta-glucans or 200 µM omeprazole for 24 h in single treatment or co-treatments. Beta-glucans from *Sacchromyces cerevisiae* were obtained from Kura srl (Torino, Italy). The experimental workflow is presented in Supplementary Figure S2.

Unless otherwise specified, reagents were purchased from Merck (Milan, Italy), whereas plasticware was from Falcon (Becton Dickinson, Franklin Lakes, NJ, USA).

### 4.2. Real-Time Polymerase Chain Reaction (qRT-PCR)

Undifferentiated and differentiated U-937 and THP-1 cells were seeded on 6-multiwell plates, and they were cultured for 24 h as reported in figure legends. The cells were washed with PBS, and total RNA was extracted with TRIzol^®^ (Invitrogen, Thermo Fisher Scientific, Waltham, MA, USA). One μg of total RNA was transcribed into cDNA using an iScript cDNA Synthesis Kit (Bio-Rad Laboratories AG, Cressier FR, Switzerland) according to the manufacturer’s instructions. The RT-PCR primers were designed with NCBI/Primer-BLAST, synthesized by Merck (Milan, Italy). Quantitative PCR was carried out in a final volume of 20 μL using the IQ™ SYBR Green Supermix (Bio-Rad Laboratories AG, Cressier FR, Switzerland) with specific primers for the quantitation of the following human genes: IL-8 (fwd 5′-GGAGAAGTTTTTGAAGAGGGCTGA-3′, rev 5′-TGCTTGAAGTTTCACTGGCATCTT-3′) [37], vacuolar ATPase (ATPase H^+^ transporting V1 subunit A (ATP6V1A) fwd 5′-TGGAGGGTGACATGGCTACTATTCAGG-3′, rev 5′-ATGGCTCCCATAATGCCAGGACCAAG-3′) [3], cytochrome *c* oxidase subunit 2 (COXII, fwd 5′-TCTGGTCAGCCCAACTCTCT-3′, rev 5′-CCTGTGATCCACCAGAAGGT-3′) [38], mitochondrial ATP synthase F0 subunit 6 (MT-ATP6, fwd 5′-CCAATAGCCCTGGCCGTAC-3′, rev 5′-CGCTTCCAATTAGGTGCATGA-3′) [38] and beta 2-microglobulin (β2M, fwd 5′-AGCAAGGACTGGTCTTTCTATCTC-3′, rev 5′-ATGTCTCGATCCCACTTAACTA-3′) [38]. PCR amplification was 1 cycle of denaturation at 94 °C for 32 min, 45 cycles of amplification including denaturation at 94 °C for 30 s and annealing/extension at 60 °C for 30 s. The quantification of each sample was carried out comparing each PCR gene product with β2M, used as a reference gene to normalize the cDNA in different samples. Data were analyzed using the 2_ΔΔCT method. Analyzed transcripts exhibited high linearity amplification plots (*r* > 0.97) and similar PCR efficiency, confirming that the expression of each gene could be directly compared. The specificity of PCRs was confirmed by melt curve analysis. Nonspecific amplifications were never detected.

### 4.3. IL-8 Quantification

After treatments, culture supernatants were centrifuged for 5 min at 10,000× *g*, and the concentrations of IL-8 were determined by enzyme-linked immunosorbent assay (ELISA), according to the manufacturer’s protocol (R&D Systems, Minneapolis, MN 55413, USA). Analyte concentrations were expressed as ng/mL supernatants.

### 4.4. Acidification Assay

The vital stain neutral red is incorporated into acidic compartments, such as lysosomes. The degree of incorporation is inversely proportional to the lysosomal pH. Untreated and treated differentiated U-937 and THP-1 cells were stained for 1 h at 37 °C in culture medium containing neutral red solution, washed three times with phosphate-buffered saline solution (PBS) and rinsed with stop buffer (1:1 of 4.02 g trisodium citrate in 153 mL H_2_O, 0.8 mL HCl 0.1 N in 86 mL H_2_O and 25 mL of 95% *v/v* methanol), as described [39]. The absorbance was read at 540 nm, and the cell acidification was evaluated by measuring the percentage of cells stained with neutral red dye versus untreated cells that were considered 100%.

### 4.5. Western Blotting Analysis

Cells were collected, washed twice in PBS and proteins were extracted by incubation in boiling sample buffer (SB: 100 mM Tris-HCl pH 6.8, 15% glycerol, 2% SDS and 1% protease inhibitor cocktail set III (Sigma Aldrich, Milan, Italy) followed by sonication. Fifty μg of total lysates were separated by 10% SDS-PAGE and analyzed by Western blotting. Membranes were incubated overnight with the mouse monoclonal antibody anti-V-ATPase B1/2 (F-6) (sc-55544, Santa Cruz, CA, USA) or monoclonal antibody anti-VDAC (31HL) (Calbiochem, La Jolla, CA, USA). After washing with 0.1% *v*/*v* PBS-Tween, the membranes were incubated for 1 h to a peroxidase-conjugated anti-mouse secondary antibody (diluted 1:5000 in 5% *w/v* PBS-Tween with milk, Bio-Rad Laboratories, Hercules, CA, United States). The membranes were washed again with PBS-Tween, and proteins were detected and quantified with a ChemiDoc^TM^ MP System (Bio-Rad Laboratories, Hercules, CA, USA). Densitometric analysis was carried out using ImageJ software (ImageJ version 1.29, Sun Microsystems Inc., Palo Alto, CA, USA). VDAC was used as internal control for protein loading, and the results of V-ATPase quantification were normalized to VDAC.

### 4.6. ROS Essay

The measure of intracellular ROS was carried out as previously described [38]. Briefly, the harvested cells were loaded for 15 min with 10 μM 2′,7′-dichlorodihydrofluorescein diacetate (DCFH-DA, Sigma Aldrich, Milan, Italy). DCFH-DA is cleaved by intracellular esterases, and it is further oxidized by ROS to form the fluorescent compound dichlorofluorescein (DCF). DCF fluorescence was determined at an excitation wavelength of 504 nm and an emission wavelength of 529 nm using a Packard EL340 microplate reader (Bio-Tek Instruments, Winooski, VT, USA). The fluorescence values were normalized to the protein content and expressed as values relative to control.

### 4.7. Statistical Analysis

Statistical analysis of data was performed using ANOVA test with Tukey’s post-hoc correction. *p* values < 0.05 were considered significant and indicated. All data were expressed as mean ±S.E.M. of three independent experiments.

## Data Availability

The data presented in this study are available within the article and supplementary material.

## Supplementary Materials

The following are available online at https://www.mdpi.com/article/10.3390/ijms22094869/s1; Figure S1: Intracellular ROS levels. Legend for Figure S1: U-937 differentiated cells were treated for 24 h, and the intracellular levels of ROS were measured. Results are expressed relative to control and as the means ±SEM. Figure S2: Experimental workflow.
