# Supplementary figures and images for: Vitamin D and Beta-Glucans Synergically Stimulate Human Macrophage Activity
Source: Int J Mol Sci. 2021 May 4;22(9):4869. doi: 10.3390/ijms22094869 (PMC8124691; doi:10.3390/ijms22094869)

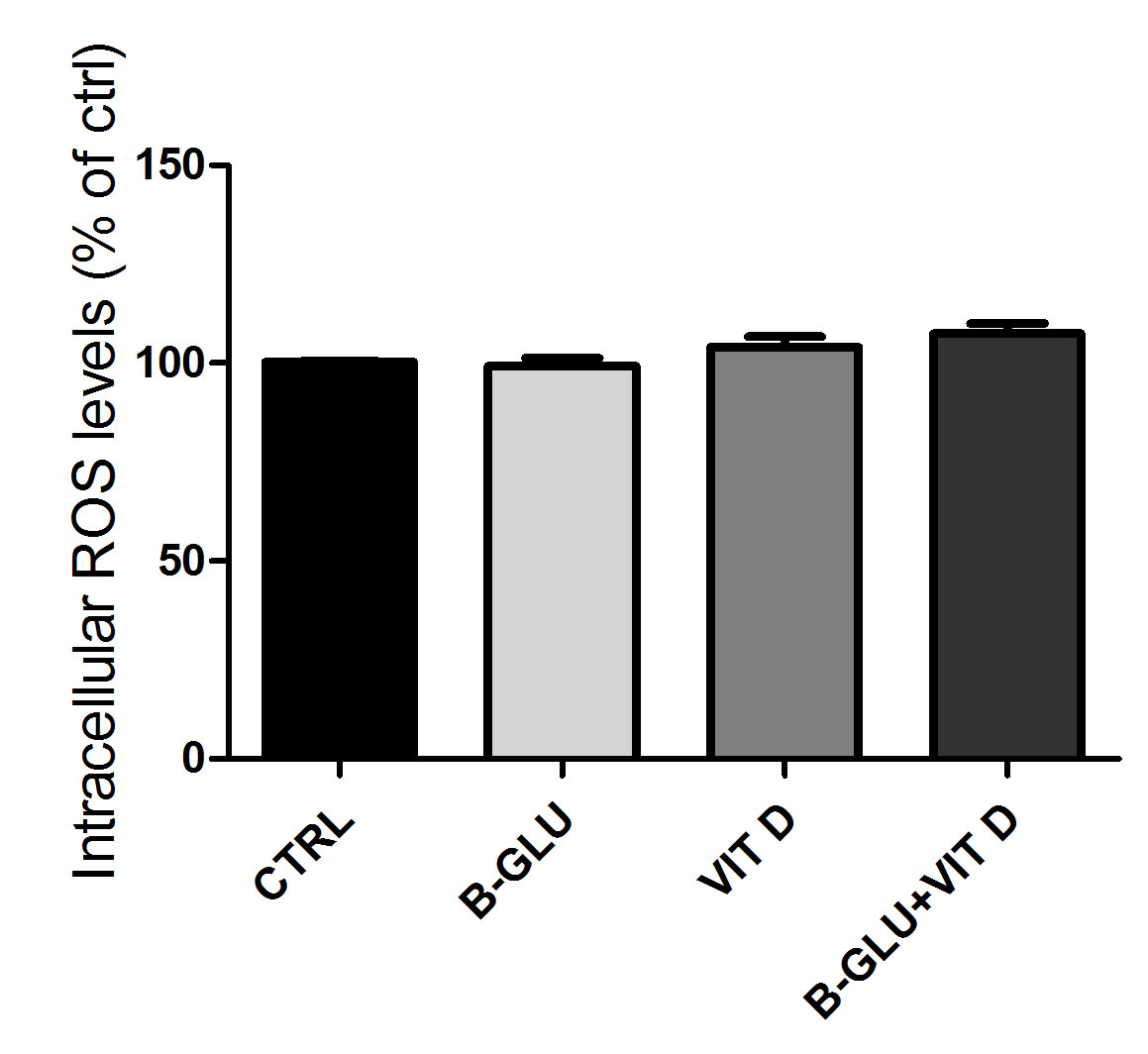

Supplement: Supplementary file 1 [file ijms-22-04869-s001.zip › Figure S1.jpg]

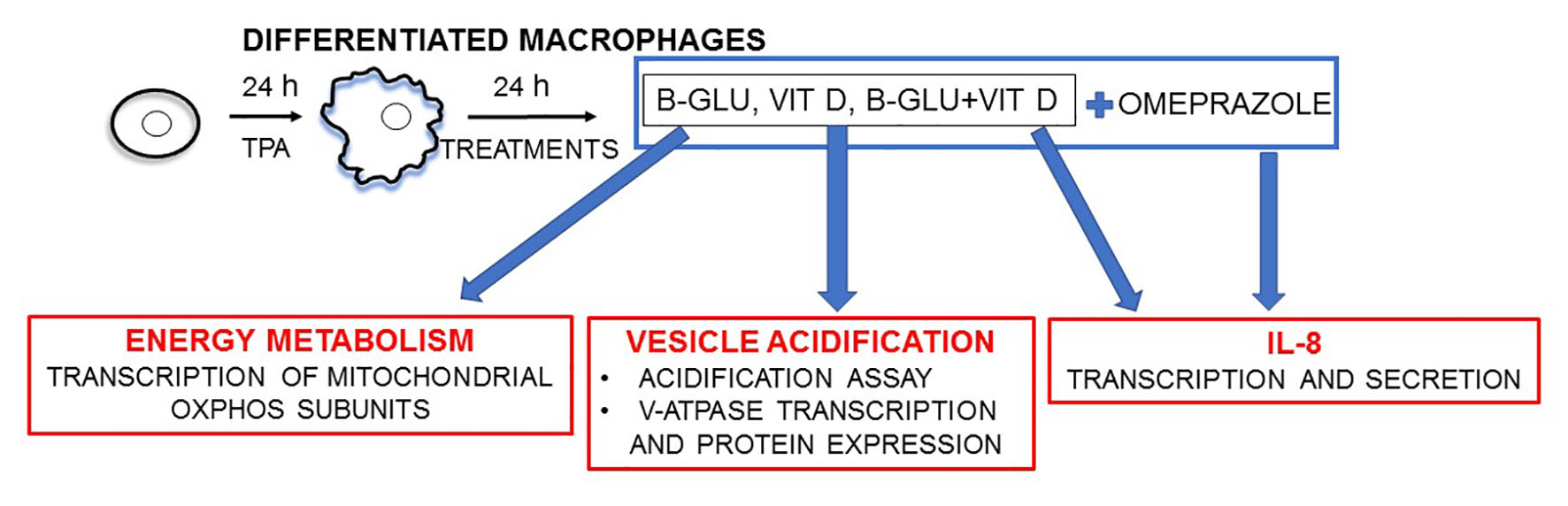

Supplement: Supplementary file 1 [file ijms-22-04869-s001.zip › Figure S2.jpg]
